# The Distribution of Boars Spermatozoa in Morphometrically Distinct Subpopulations after In Vitro Exposure to Radiofrequency Electromagnetic Radiation at 2500 MHz and Their Motility

**DOI:** 10.3390/ani14060828

**Published:** 2024-03-07

**Authors:** Ivona Žura Žaja, Silvijo Vince, Ivan Butković, Kim Senaši, Nina Poljičak Milas, Krešimir Malarić, Martina Lojkić, Ivan Folnožić, Suzana Milinković Tur, Mario Kreszinger, Marko Samardžija, Snježana Čipčić, Nikolino Žura, Mario Ostović, Marinko Vilić

**Affiliations:** 1 Department of Physiology and Radiobiology, Faculty of Veterinary Medicine, University of Zagreb, 10 000 Zagreb, Croatia; izzaja@vef.unizg.hr (I.Ž.Ž.); tur@vef.unizg.hr (S.M.T.); mvilic@vef.unizg.hr (M.V.); 2 Clinic of Obstetrics and Reproduction, Faculty of Veterinary Medicine, University of Zagreb, 10 000 Zagreb, Croatia; svince@vef.hr (S.V.); mlojkic@vef.unizg.hr (M.L.); folnozic@vef.unizg.hr (I.F.); smarko@vef.unizg.hr (M.S.); 3 Faculty of Veterinary Medicine, University of Zagreb, 10 000 Zagreb, Croatia; kim.senasi996@gmail.com; 4 Department of Pathological Physiology, Faculty of Veterinary Medicine, University of Zagreb, 10 000 Zagreb, Croatia; nmilas@vef.unizg.hr; 5 Department of Communication and Space Technologies, Faculty of Electrical Engineering and Computing, University of Zagreb, 10 000 Zagreb, Croatia; kresimir.malaric@fer.hr; 6 Clinic of Surgery, Orthopedics and Ophthalmology, Faculty of Veterinary Medicine, University of Zagreb, 10 000 Zagreb, Croatia; 7 University of Applied Health Sciences, 10 000 Zagreb, Croatia; bielienoci@gmail.com (S.Č.); nikolino.zura@zvu.hr (N.Ž.); 8 Department of Animal Hygiene, Behaviour and Welfare, Faculty of Veterinary Medicine, University of Zagreb, 10 000 Zagreb, Croatia; mostovic@vef.unizg.hr

**Keywords:** boars, radiofrequency electromagnetic radiation, exposure, morphometric analysis, spermatozoa subpopulations

## Abstract

**Simple Summary:**

The global use of anthropogenic radiofrequency electromagnetic radiation (RF-EMR) in wireless technologies is increasing exponentially and presents a potential risk to animals, especially domestic animals and pets. Additionally, the semen of boar is, in the process of collection, manipulation and storage for the artificial insemination (AI) of sows, surrounded by and exposed to these different sources of wireless technologies devices. A frequency of 2.5 GHz (this frequency band is used in 5G technology) is of particular interest because many studies have used the frequency bands of 4G technology. For the efficiency of pig production and breeding, it is extremely important to determine the effects of such radiation on semen quality and sow fertilization success. Therefore, we aimed to investigate the effect of RF-EMR at 2500 MHz on in vitro exposed breeding boar semen spermatozoa motility and the proportions of spermatozoa subpopulations according to morphometric parameters. The progressive spermatozoa motility and the proportion of the spermatozoa subpopulation with a higher fertilizing potential were significantly reduced in the experimental group. These results indicate the importance of further research on the effects of RF-EMR on different animal species, especially in those undergoing AI procedures, which are important both in terms of the quality of semen and fertilization and production and breeding goals.

**Abstract:**

Anthropogenic radiofrequency electromagnetic radiation (RF-EMR) from wireless technologies has increased dramatically. The boar semen used for artificial insemination is essential in sustaining the pig industry, and additionally it is also exposed to the effects of the RF-EMR of wireless technologies. Furthermore, there are no data on the effects of RF-EMR on semen quality, and this is the first analysis of sperm’s morphometric parameters for assessing the effect of RF-EMR on the spermatozoa subpopulations of boars. This study investigated the effect of RF-EMR on in vitro exposed breeding boar semen spermatozoa motility and the proportions of spermatozoa subpopulations according to their morphometric head and tail parameters. The semen samples of 12 boars were divided into control and experimental groups. The samples in the experimental group were exposed in a gigahertz transverse electromagnetic chamber at a frequency of 2500 MHz (the frequency band used in 5G technology) and an electric field strength of 10 Vm^−1^ for two hours. After exposure, the spermatozoa motility was evaluated for both groups. A morphometric analysis of the semen smears was performed using SFORM software (Version 1.0; VAMS, Zagreb, Croatia). The progressive spermatozoa motility was significantly reduced in the experimental group (74.7% vs. 85.7%). PC analysis and cluster analysis revealed two spermatozoa subpopulations: S1, spermatozoa with a more regular head shape and a smaller midpiece outline, and S2, spermatozoa with a more elongated head shape and a larger midpiece outline. The experimental semen samples had a greater proportion of the S1 spermatozoa subpopulation (68.2% vs. 64.4%). The effect of RF-EMR at 2500 MHz on the in vitro exposed boar semen resulted in decreased progressive spermatozoa motility and a lower proportion of the spermatozoa subpopulation with a higher fertilizing potential.

## 1. Introduction

An enormous surge in wireless communication, with the consequent increase in human and animal exposure to radiofrequency electromagnetic radiation (RF-EMR), has been evident in recent decades. Based on many studies, there is sufficient evidence that RF-EMR of anthropogenic origin has increased many times over in nature and that this radiation affects the environment. For example, anthropogenic radiofrequency electromagnetic radiation from wireless technologies has increased the natural levels of the around 1 GHz frequency band in nature by about 10^18^ times [1]. Therefore, RF-EMR at today’s intensity is known as “electro-pollution”. Wi-Fi-based technology and receivers, such as laptops, tablets and mobile phones with their base stations, as well as Bluetooth devices, are now routinely used [2,3,4]. Although such technology has significantly improved our quality of life, it cannot be ruled out that it is also the cause of many ailments and diseases. The harmful effect of RF-EMR can be manifested in most organ systems; however, one of the most sensitive organ systems is the male reproductive system [3,4]. It is well known that RF-EMR reduces the quality of semen and has genotoxic effects on humans and animals both in vitro and in vivo [5,6,7]. The exposure of men to RF-EMR through various devices, such as mobile phones, wireless internet and laptops, causes abnormal spermatozoa morphology, a decrease in spermatozoa count due to apoptosis, reduced spermatozoa motility and viability, increased testosterone levels, decreased luteinizing hormone levels and increased spermatozoa DNA fragmentation [8,9,10,11,12,13,14]. These effects are correlated with the time of exposure [15]. A decrease in semen quality in humans (a decrease in progressive spermatozoa motility and an increase in spermatozoa DNA fragmentation) is also evident when the semen is exposed ex vivo to a laptop connected to a wireless network, i.e., a Wi-Fi frequency of 2.4 GHz for 4 h [16]. Moreover, an increased percentage of damaged epididymal spermatozoa heads was found in rats exposed to 24 h RF-EMR at a frequency of 2.4 GHz for a year [17]. The biological targets of RF-EMR are cell structures such as the plasma membrane (causing cell membrane permeability, including changes in calcium levels, ionic distribution and ion permeability), mitochondria and DNA [5,18]. Spermatozoa motility and morphology are important characteristics to assess in determining semen quality [19]. The introduction of computer-assisted sperm analysis (CASA) has advanced quality assessments of human and animal semen and the diagnosis of fertility, enabling the assessment of parameters such as motility and morphology [19]. The current computer-assisted sperm morphometric analysis (ASTMA) can be used to more accurately analyze individual spermatozoa morphometrics [20,21]. By applying ASTMA technology and multivariate procedures such as cluster analysis, it was observed that boar semen samples contained spermatozoa subpopulations of different morphometric characteristics that are not detectable by conventional subjective methods [22,23]. Morphometric results can vary depending on internal factors such as individual variability, species, breed, sexual maturity and age [23,24,25], and external factors including environmental factors, sample preparation and the morphometric analysis of semen [20,26]. 

To date, there are no data on the effects of RF-EMR on the reproductive system of domestic animals. Moreover, it cannot be ignored that the environment surrounding boars at farms is subject to constant increases in radiofrequency electromagnetic fields from different sources, including mobile phone base stations and wireless communication devices. Therefore, for the efficiency of pig production and breeding, it is extremely important to determine the effects of such radiation on the reproductive system of boars. The aim of this study was to investigate the effect of RF-EMR at 2500 MHz (the frequency band used in 5G technology), since it is very close to 2.45 GHz, the frequency of wireless communication devices (Wi-Fi and Bluetooth), on in vitro exposed breeding boar semen spermatozoa motility and proportions of spermatozoa subpopulations using principal component (PC) and cluster analyses of morphometric head and tail parameters.

## 2. Materials and Methods

### 2.1. Animals, Housing and Feeding

The study included a total of 12 boars of the Pietrain (8) and German Landrace (4) breeds, aged 1.5–3.5 years, from which semen was routinely taken twice a week, on a certain day of the week for each boar for the artificial insemination of sows. The boars are owned by the Centre for Artificial Insemination, Stočar d.o.o., Varaždin, Croatia. The boars were housed individually in 12 m^2^ (4 × 3 m) pens, with straw bedded floors and natural lighting, and were fed twice a day, around 6:00 a.m. and 2:00 p.m., with a mixture produced by Stočar d.o.o., Varaždin, Croatia. The daily requirements of the boar are approximately 2 kg of mixture, with the following composition: crude protein (17.15%), crude fat (3.13%), crude cellulose (6.29%), ash (5.30%), metabolic energy (12.49 MJ/kg), lysine (1.24%), methionine (0.47%), tryptophan (0.18%), methionine + cystine (0.76%), zinc (126 mg/kg), magnesium (56 mg/kg), digestible protein (38.28%), copper (31.50 mg/kg), selenium (0.42 mg/kg), calcium (0.83%), total phosphorus (0.53%), usable phosphorus (0.22%), sodium (0.23%), vitamin D3 (1750.01 IU/kg), vitamin A (9100 IU/kg) and vitamin E (70 IU/kg).

### 2.2. Collection and Evaluation of Semen from the Boars

Collection of semen samples was performed in the morning hours (around 7:00 a.m.). The procedure for obtaining the ejaculate was carried out by the method of manual fixation of the penis. Standard evaluation of boar ejaculate was performed at the Centre for Artificial Insemination, Stočar d.o.o., Varaždin, Croatia. The semen was collected in wide-mouthed glass containers. The semen volume was determined using a measuring cup, and the concentration of sperm in the ejaculate was determined using an Accucell photometer type 60CI0394 (IMV technologies, Normandy, France). Mass motility was determined in native semen using an Olympus BX50F (Olympus, Tokyo, Japan) microscope with a built-in spermotherm. After determining sperm concentration and mass motility, a certain amount of Cronos diluent (Medi-Nova, Reggio Emilia, Italy) was added to the semen and it was transferred to 80 mL plastic bottles. The samples of semen were transported in specialized containers with thermometer (to protect against the harmful effects of light and temperature) for 45 min from the collection place to the laboratory. 

### 2.3. Exposure of Samples to RF-EMR in Laboratory Conditions 

**Figure 1:**
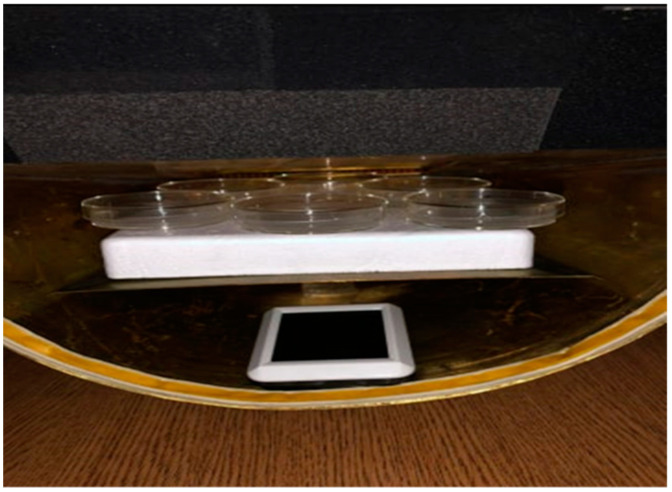
Gigahertz transverse electromagnetic (GTEM) chamber. View of the GTEM chamber in which the boar semen samples were placed together with a digital thermometer.

Upon arrival at the clinic, samples of each boar (*n* = 12) were divided into two Petri dishes (control and experimental sample (12 each for a total of 24 samples). The experimental samples were exposed in a gigahertz transverse electromagnetic (GTEM) chamber to RF-EMR at a frequency of 2500 MHz and an electric field strength of 10 Vm^−1^ for two hours. GTEM was located at the Clinic for Reproduction and Obstetrics at the Faculty of Veterinary Medicine University of Zagreb (45°48′25.91″ North and 16°0′20.49″ East). A GTEM-chamber was made at the Department of Communication and Space Technologies, Department of Radiocommunications and High-Frequency Electronics, Faculty of Electrical Engineering and Computing, University of Zagreb, Zagreb, Croatia. The chamber contained a digital thermometer to measure temperature to determine if there was a thermal effect of the radiation (Figure 1). The average temperature inside the chamber during semen exposure was 19.1 °C (range 18.7–19.5 °C). Control samples (unexposed samples) were placed in a metal container (imitation for GTEM-chamber) and kept under the same conditions (temperature and time) as the experimental groups but without exposure to RF-EMR. Experimental samples (exposed) and control samples (unexposed) were assessed after exposure/sham-exposure of 2 h. In addition to the chamber, an HP 8657A signal generator and an RFGA0101-05 linear amplifier were used to achieve electromagnetic field strength. The amplifier was connected to a computer and the desired frequency was set using the SynthNV program (Windfreak Technologies, LLC., New Port Richey, FL, USA). The GTEM-chamber is a transmission line that is based on TEM-chambers where the letter G indicates that the GTEM chamber works in the GHz range up to 18 GHz. The GTEM-chamber is adjustable in the pyramidal part of the TEM-chamber with an impedance of 50 Ω. After exposure, the control and experimental semen samples were evaluated for spermatozoa motility.

### 2.4. Computer-Assisted Sperm Analysis

Spermatozoa motility was determined using a computer-assisted sperm analysis (CASA) device (Integrated Visual Optical System, Version 12; Hamilton Thorne Research, Beverly, MA, USA) located at the Clinic of Obstetrics and Reproduction, Faculty of Veterinary Medicine, University of Zagreb, Zagreb, Croatia. After the exposure procedure of the experimental samples, diluted experimental and control semen samples (5 μL each) were applied to a 20 μm deep Leja-chamber (Leja Products B.V., Nieuw Vennep, The Netherlands) and placed on a heated spermotherm (Minitub, Tiefenbach, Germany). After the cessation of passive spermatozoa movement, imaging was performed on all eight fields of the chamber. The program was set to analyze 45 frames obtained per field at a frame rate of 60 Hz. The analysis determined spermatozoa motility (%) and progressive motility (%). 

### 2.5. Preparation and Staining of Semen Smears

The control and experimental semen samples were then used to make a smear on a glass slide. The semen smears were then stained with the Spermac set of reagents (Minitube, Tiefenbach, Germany) for diagnostic staining of spermatozoa, which is generally used to visualize the head, acrosome, equatorial region, central part and tail of spermatozoa. The Spermac method in brief: a thin smear was made on a clean slide, then placed in fixative for 5 min. After fixation, the slides were placed on a heating plate at 37 °C for 15 min. Then the slides were washed with distilled water and staining followed by first immersing the smear in 50 mL of red liquid (Spermac “A”) for 1 min, followed by rinsing again with distilled water; afterwards, the smear was immersed in 50 mL of pale green liquid (Spermac “B”) for 1 min, after which the smear was rinsed with distilled water and the last smear was immersed in 50 mL of dark green liquid (Spermac “C”) for 1 min, with a final rinse. After staining, the final drying of the preparation followed on a heating plate at a temperature of 37 °C.

### 2.6. Morphometric Analysis of Spermatozoa

In total, 24 stained semen smears were analyzed, with approximately 70 spermatozoa measured for each smear (*n* = 1691). The spermatozoa morphometric analysis was performed using the SFORM program for image processing and analysis (VAMSTEC, Zagreb, Croatia). Only spermatozoa heads that did not overlap with those of other spermatozoa and non-banded tails were measured and analyzed. The borders of the head, midpiece and tail of the spermatozoa were marked automatically using the marking option of SFORM (first the head, then the midpiece and finally the tail) with manual correction using a computer mouse and then the calculated data were printed in the program table [27]. The area (μm^2^), outline (μm), minimum radius (μm), maximum radius (μm), length (μm) and width (μm) were the primary morphometric parameters (size parameters) calculated for the spermatozoa head and midpiece, while for spermatozoa tail, only length was calculated. Different ratios of morphometric parameters were also calculated, such as the total length of the spermatozoa, which is the sum of the head length and the tail length, then head length/total length, head length/tail length, tail length/total length, head outline/total length, head area/total length and head length × head width/total length. Some primary morphometric parameters (head size morphometric parameters) were used to calculate head shape morphometric parameters using the following formulas: ellipticity (length/width), rugosity (4π × area/outline2), elongation (length − width)/(length + width) and regularity (π × length × width/4 × area).

### 2.7. Statistical Data Processing

A statistical data analysis was performed using the SAS 9.4 software package (Statistical Analysis Software 2002–2012 by the SAS Institute Inc., Cary, NC, USA). A descriptive data analysis was performed using MEANS and FREQ procedures. The dependent parameters between groups were analyzed via a multivariate analysis of variance (MANOVA) based on Wilks’ lambda criterion using the GLM procedure. The results are expressed as least squares means (LSM) and 95% confidence intervals. To compare mean values, the TukeyKramer method of multiple comparisons was used at the level of statistical significance *p* < 0.05. 

**Figure 2:**
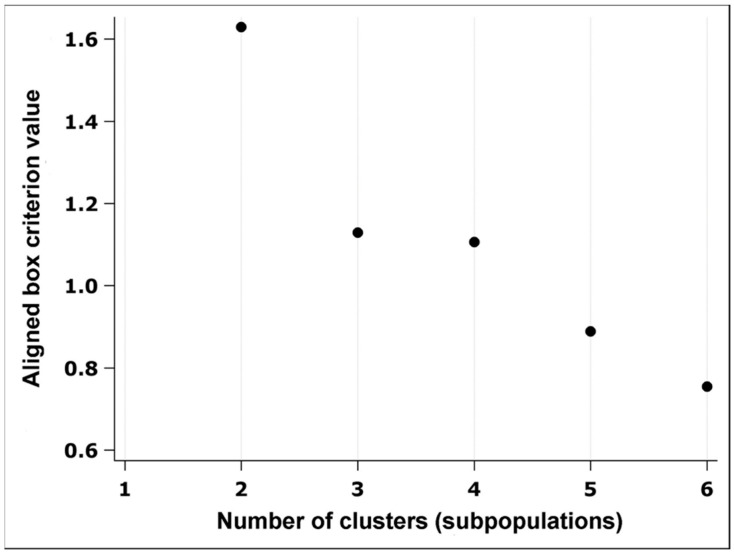
Two subpopulations of boar spermatozoa. The figure shows that two optimal subpopulations were obtained using the values of the equalized box criterion.

Multivariate clustering analyses (CLUSTERS) of data were performed through several steps to obtain spermatozoa subpopulations based on the data of the main morphometric parameters of the spermatozoa head and tail. The first step was the analysis of the main components (PROC FACTOR) to obtain the characteristic values (eigenvalues) of the morphometric parameters using the Kaiser criterion (λ ≥ 1) to determine the number of main components. The number of clusters in the K-means cluster analysis was determined using the HPCLUS procedure, which selects the best k value (number of clusters or subpopulations) using the aligned box criterion value (Figure 2). The third step was to group the data using non-hierarchical analysis (K-means method and Euclidean distance) of the most important parameters for each component from the previous analysis using the FASTCLUS procedure. In order to better interpret the data of the obtained spermatozoa subpopulations, stepwise discrimination analysis (PROC STEPDISC) and testing of atypical values (PROC FASTCLUS) were performed. Testing for differences in the distribution of spermatozoa subpopulations between the control and experimental semen sample groups was done using Chi-square and Mantel-Haenszel Chi-square tests (PROC FREQ).

## 3. Results

### 3.1. Overall Semen Variables

Standard evaluation of ejaculate revealed that the semen samples correspond to the criteria valid for boars (minimum motility > 60%) and in this respect the conditions were met since average spermatozoa motility was about 80%. The mean volume (±SD) of the boar semen sample was 354.5 ± 126.3 mL. Sperm concentration in mL of semen (±SD) was 358.7 ± 169.4 million. The total sperm concentration (±SD) was 113.5 ± 34.6 billion.

### 3.2. Individual Morphometric Parameters of the Spermatozoa Head and Tail

Morphometric analysis was performed on a total of 1691 spermatozoa, of which 839 spermatozoa (49.6%) in the control sample and 852 (50.4%) in the experimental group. Statistical analysis of individual spermatozoa morphometric parameters between the experimental group exposed to RF-EMR at a frequency of 2500 MHz and the control group (Table 1, Table 2 and Table 3) revealed no statistically significant differences. The only morphometric parameter whose value was close to statistical significance was the midpiece convex (*p* = 0.06). Sperm motility determined by CASA between the experimental and control groups was not statistically significantly different, although motility was reduced in the experimental group (74.7%) as compared to the control group (85.7%). Progressive sperm motility was statistically significantly reduced (*p* < 0.001) in the experimental group (35.0%) compared to the control group (60.1%).

### 3.3. Spermatozoa Subpopulations Based on Morphometric Parameters of Spermatozoa Head and Tail

Analysing the main components before grouping, four components (factor 1, 2, 3 and 4) with a characteristic value (λ ≥ 1) were retained. All four components in total explained 84.6% of the variance of the morphometric parameters of the spermatozoa head, midpiece and tail (Table 4).

Using Table 4, the most important parameters were selected from each component (head length, head width, midpiece outline and regularity of the spermatozoa head). The final number of subpopulations was obtained using the value of the equalised box criterion. This analysis determined that the two subpopulations are the most optimal because the value of the equalised box criterion was the highest (Figure 2).

The grouping analysis revealed well-defined differences between head length, width and regularity and the midpiece outline in the two spermatozoa subpopulations (S1 subpopulation—smaller length and larger head width, more regular head shape and smaller midpiece outline, and S2 subpopulation—longer length and smaller head width, more elongated head shape and larger midpiece outline) (Table 5). 

**Figure 3:**
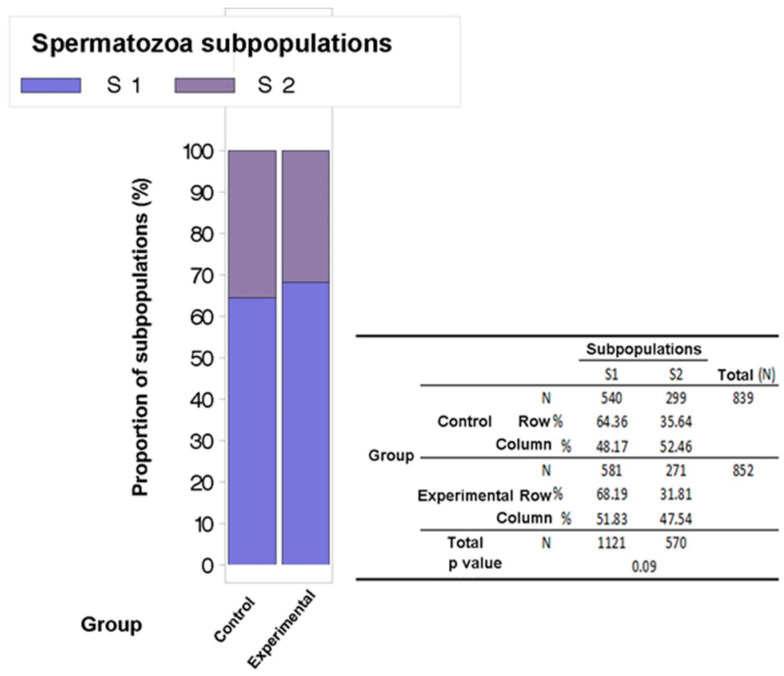
Proportion of spermatozoa subpopulations in control and experimental semen samples groups.

Statistical analysis of the obtained subpopulations between the control and experimental groups of semen samples revealed that the experimental semen samples group had a higher percentage of spermatozoa of the S1 subpopulation (68.2% vs. 64.4%) and a lower percentage of the S2 subpopulation (31.8% vs. 35.6%) which is close to statistical significance (*p* = 0.09) (Figure 3). 

## 4. Discussion

This study showed that the exposure of semen of breeding boars in vitro to RF-EMR at a frequency of 2500 MHz and an electric field strength of 10 V/m for a duration of 2 h did not cause changes of spermatozoa individual morphometric parameters and sperm motility, but it decreased progressive sperm motility.

Although it is known that RF-EMR has a harmful effect on the male reproductive system, by reducing the number of Leydig cells, motility, and number of spermatozoa, and altering spermatozoa morphology in humans and animals [9,15,28,29,30,31,32], the results presented here do not support this. This could be due to the application of different study designs (protocols) during the experiment, different species and ages of animals, and different analysis methods. Furthermore, to the extent of our knowledge, the effect of RF-EMR on spermatozoa morphometric parameters has not previously been investigated in humans or other species. In addition, spermatozoa morphometry in boars is performed using different software and on semen smears stained with different methods. Wysokińska et al. [33] performed spermatozoa morphometric analysis on spermatozoa samples collected from 35 boars of the Polish Landrace breed at the age of 7 to 8 months stained with the Bydgoszka method using a computer image analysis package (Screen Measurement v. 4.1, Laboratory Imaging S.r.o. LIM, Prague, Czech Republic). Their research showed a lower mean length, width, area and outline of the head and a higher mean value for the total length and tail length compared to the results for the control group in the present study. This study used the computer program SFORM (VAMSTEC, Zagreb, Croatia) for the morphometric analysis, and there is no information about its use in the morphometric analysis of boar spermatozoa in the literature, and therefore, it is possible that the difference in the stated values was result of the differences in the programs for morphometric analysis or differences in staining methods, age and breed of boars. Górski et al. [34] showed a lower mean value for the length, width, area and outline of the spermatozoa head and a higher mean value for the spermatozoa total length and the tail length as compared to the control group of this study. Those authors performed a spermatozoa morphometric analysis on semen samples collected from 12 boars of the Duroc breed, stained using the method according to Kondracki et al. [24], and using the computerised image analysis system Screen Measurement v. 4.1. for morphometric measurements. One reason for the differences between the results of our control group and that study could be due to the different staining methods [26], animal breed [23] or the analysis method used. 

Other aspects of spermatozoa physiology and morphology may also need to be considered, as they may affect their ability to actively move through the female reproductive system. In many species, the first barrier is the cervical mucus, which allows only progressively motile spermatozoa with normal morphology to pass into the uterus and through which they progressively move (with the help of myometrial contractions) to the fallopian tube, where fertilization occurs [35]. Therefore, spermatozoa motility is crucial. This study showed a significant reduction in progressive spermatozoa motility after exposure of the semen of the boars in vitro to RF-EMR at a frequency of 2500 MHz. Mailankot et al. [10] reported similar results, showing that exposing rats to RF-EMR 1 h a day for 28 days at a frequency of 900 and 1800 MHz originating from mobile devices caused a drop in the number of motile spermatozoa, and reduced progressive sperm motility. Oni et al. [36] and Gorpinchenko et al. [37] investigated the effect of electromagnetic radiation at the frequency of mobile telecommunications (900/1800 MHz) and laptops (2.45 GHz) on in vitro samples of human semen, and found that exposure to these frequencies also reduced the number of motile and progressively motile spermatozoa. On the other hand, some studies have reported no changes in spermatozoa motility after rats had been long-term exposed to RF-EMR frequency of 2.4 GHz [17]. A possible mechanism that leads to a decrease in spermatozoa motility after exposure of semen to RF-EMR is a lowered mitochondrial potential or oxidative stress, and consequently impaired spermatozoa vitality [28,38]. Namely, some studies on the impact of RF-EMR on spermatozoa, though not on boars, have indicated that RF-EMR at the frequency of mobile telephony can cause the formation of reactive oxygen species (ROS) and thus oxidative stress. It is also known that in aerobic organisms, a balance between antioxidant processes and reactive compounds formed requires an oxidative-reduction balance, because otherwise, during oxidative stress, an excess of ROS leads to the damage of numerous molecules. Kumar et al. [39] and Meena et al. [40] showed that exposing rats to RF-EMR frequencies of 2.45 GHz and 10 GHz for 2 h a day for 45 days led to cell damage mediated by oxidative stress, i.e., caused an increase in the concentration of ROS, an increase in the percentage of spermatozoa apoptosis in testicles, and DNA damage.

This study has shown that RF-EMR at a frequency of 2500 MHz could have a negative effect on the success of egg fertilisation. We base the above assumption not only on the obtained results that RF-EMR reduces the number of progressively motile spermatozoa, but also on the obtained percentages of the spermatozoa subpopulation after exposure to RF-EMR. The obtained proportions of spermatozoa subpopulations using PC and cluster analysis according to morphometric head and tail parameters showed that the experimental group of semen had a higher percentage of the less desirable subpopulation, characterised by spermatozoa of smaller length and larger head width, more regular head shape and smaller midpiece outline as compared to the more desirable spermatozoa characterised by longer length and larger head width, more elongated shape of the head and larger midpiece outline. It has been empirically proven in different species that spermatozoa function is related to its morphometry, which includes the area of the head, midpiece and tail [41,42]. Ramon et al. [43] stated that deer ejaculate containing a high percentage of spermatozoa with fast and linear motility have small and elongated heads and achieve higher fertility. An elongated spermatozoa head can have an important function in that such sperm will be hydrodynamically more efficient due to less resistance in forward movement, which can affect the fertilising ability of sperm [20,44]. However, Barquero et al. [23] reported that boars with a larger litter size had significantly less elongated spermatozoa, and the mortality of piglets was greater in these males. Evolutionary biology is still debating which of the two spermatozoa components, head characteristics or midpiece traits of the spermatozoa, is more important in spermatozoa competition during the egg fertilisation process. Namely, the increase in the midpiece of the spermatozoa increases its energy due to the increased area housing the mitochondria [45], as more energy is needed for faster sperm. FIRMAN and SIMMONS [46] reported that midpiece size is a predictor of swimming speed of Mus musculus domesticus spermatozoa. It is known that RF-EMR can cause cell/sperm apoptosis, and mitochondria are the main initiators of apoptosis [47]. In addition, RF-EMR promotes increased mitochondrial ROS production and expression of mitochondrial apoptotic markers [48] with decreased mitochondrial membrane potential [47,49]. Although the exact mechanism of apoptotic changes in spermatozoa, and in somatic cells, is still unknown, one of the signs of apoptosis was described as typical cell shrinkage [50]. The decrease in the mitochondria outline with reduced progressive motility of spermatozoa of the experimental group in this study could indicate the initiation of spermatozoa apoptosis. Furthermore, exposure of cells to RF-EMR if the intensity of the fields increases beyond the threshold, causes electroporation, during which water pores are created in the membrane, disrupting the ion balance and leading to water ingress in the cell [18], which is likely to cause a change in spermatozoa shape in a higher proportion in the experimental group in this study. If this was the case, then the increased ROS production generated in these highly vulnerable cells could reasonably be expected to impose an oxidative stress environment upon the aforementioned the sperm population. Given that there is no literature on the effects of RF-EMR on the proportion of boars or other species spermatozoa subpopulations obtained based on morphometric parameters, the results of the present study cannot be compared. There are scarce studies on the proportions of boar spermatozoa subpopulations using PC and cluster analysis according to morphometric parameters, therefore it is not possible to compare the obtained results. However, Barquero et al. [23] investigated the spermatozoa morphometry of boars without exposure to RF-EMR, and found four subpopulations of spermatozoa using PC and cluster analysis according only to morphometric head parameters: subpopulation 1 with lower values for ellipticity and the widest heads, sub-population 2 with the highest values for head area and perimeter, subpopulation 3 with shorter length, with a smaller area and head perimeter values, and subpopulation 4 with the highest values for head length, ellipticity, and elongation. We obtained two subpopulations related to not only morphometric head but also tail parameters, giving a more complete picture of spermatozoa morphology, which is related to their function, i.e., fertilisation ability.

## 5. Conclusions

The effect of RF-EMR at 2500 MHz on in vitro exposed breeding boar semen for two hours was seen in the decreased progressive spermatozoa motility and proportion of the spermatozoa subpopulation with a more elongated head shape and larger midpiece outline. Further research on the effects of RF-EMR on different animal species and breeds, especially domestic animals, is important both for the quality of semen and fertilization and for production and breeding goals. The observed results could also be crucial for comparison with human reproductive medicine and potential adverse effects during the specific technological process of semen processing on breeding pigs farms.

## Figures and Tables

**Table 1:** Boar spermatozoa morphometric head parameters in the control and experimental semen sample groups.

Spermatozoa Morphometric Parameters		Control Group		Experimental Group		
		Mean	95% Confidence Interval	Mean	95% Confidence Interval	*p* Value
Morphometric parameters of the head	Area (µm^2^)	46.79	46.47–47.11	46.75	46.43–47.07	0.84
	Outline (µm)	27.31	27.21–27.41	27.26	27.16–27.36	0.49
	Minimal radius (µm)	2.48	2.47–2.49	2.48	2.47–2.49	0.81
	Maximal radius (µm)	5.34	5.32–5.37	5.34	5.32–5.37	0.93
	Convex (µm)	47.39	47.06–47.72	47.43	47.11–47.76	0.85
	Length (µm)	10.31	10.26–10.36	10.3	10.26–10.35	0.86
	Bredth (µm)	5.45	5.42–5.47	5.45	5.42–5.47	0.89
	Ellipticity	1.9	1.89–1.91	1.89	1.89–1.91	0.78
	Rugosity	0.79	0.785–0.789	0.79	0.787–0.791	0.27
	Elongation	0.31	0.305–0.311	0.31	0.30–0.31	0.8
	Regularity	0.94	0.941–0.944	0.94	0.94–0.95	0.26

Ellipticity = length/bredth; Rugosity = (4π × area/outline2); Elongation = [(lenght − bredth)/(length + bredth)]; Regularity = (π × length × bredth/4 × area).

**Table 2:** Boar spermatozoa morphometric midpiece and tail parameters in the control and experimental semen sample groups.

Spermatozoa Morphometric Parameters		Control Group		Experimental Group		
		Mean	95% Confidence Interval	Mean	95% Confidence Interval	*p* Value
Parameters of morphometric characteristics of the midpiece and the tail	Midpiece area (µm^2^)	19.42	19.29–19.55	19.48	19.35–19.60	0.52
	Midpiece outline (µm)	30.44	30.32–30.56	30.35	30.23–30.46	0.27
	Midpiece min. radius (µm)	0.39	0.37–0.39	0.39	0.38–0.40	0.66
	Midpiece max. radius (µm)	6.78	6.75–6.81	6.76	6.73–6.78	0.25
	Midpiece convex (µm)	24.58	24.32–24.84	24.92	24.67–25.18	0.06
	Midpiece length (µm)	13.25	13.19–13.30	13.2	13.15–13.26	0.27
	Midpiece width (µm)	2.05	2.03–2.08	2.08	2.05–2.11	0.21
	Tail length (µm)	34.05	33.84–34.26	33.92	33.71–34.12	0.37

**Table 3:** Boar spermatozoa morphometric head and tail parameters ratios in the control and experimental semen sample groups.

Spermatozoa Morphometric Parameters		Control Group		Experimental Group		
		Mean	95% Confidence Interval	Mean	95% Confidence Interval	*p* Value
Different ratios of morphometric parameters	Total length *	44.36	44.13–44.59	44.22	43.99–44.45	0.4
	Head length/Total length	0.23	0.232–0.234	0.23	0.23–0.24	0.52
	Head length/Tail length	0.31	0.30–0.31	0.31	0.30–0.31	0.53
	Tail length/Total length	0.77	0.766–0.768	0.77	0.765–0.767	0.52
	Head outline/Total length	0.62	0.615–0.621	0.62	0.616–0.621	0.78
	Head area/Total length	1.06	1.05–1.06	1.06	1.05–1.07	0.75
	Head length and width/ Total length *	1.27	1.26–1.28	1.27	1.27–1.28	0.53

* Total length = head length + tail length.

**Table 4:** Eigenvalues of boar spermatozoa morphometric head and tail parameters in the analysis of the main components. Four components (factor 1, 2, 3, 4) with a characteristic root λ ≥ 1 were retained—Kaiser’s criterion.

Spermatozoa Indicators	Factor 1	Factor 2	Factor 3	Factor 4
Head length	0.93 *			
Head width		0.97 *		
Head area	0.62			
Head outline	0.83			
Ellipticity	0.72			
Rugosity	−0.69			
Elongation	0.72			
Regularity				0.89 *
Midpiece length			0.70	
Midpiece width		0.39		
Midpiece area		0.65		
Midpiece outline			0.72 *	
Tail length	0.39			
Characteristic root (λ) and explained variance (%)	4.78 (36.8)	3.66 (28.2)	1.53 (11.8)	1.01 (7.8)

* The most important parameters for each factor.

**Table 5:** Subpopulations of boar spermatozoa (S1 and S2) obtained using the analysis of grouping of spermatozoa morphometric head and midpiece parameters.

	Spermatozoa Subpopulation	
Spermatozoa Morphometric Head and Midpiece Parameters Mean ± SD	S1	S2
*n* (%)	942 (55.71)	749 (44.29)
Head length (µm)	10.16 ± 0.65	10.77 ± 0.68
Head width (µm)	5.44 ± 0.38	5.45 ± 0.35
Midpiece outline (µm)	29.67 ± 1.17	32.66 ± 1.09
Regularity	0.943 ± 0.02	0.941 ± 0.02

S1—Spermatozoa of smaller length and larger head width, more regular head shape and smaller midpiece outline; S2—Spermatozoa of longer length and smaller head width, more elongated head shape and larger midpiece outline.

## Data Availability

The data presented in this study are available on reasonable request from the corresponding author. The data are not publicly available due to planned research in the future.
